# The extracellular matrix protein fibronectin promotes metanephric kidney development

**DOI:** 10.1007/s00424-024-02954-9

**Published:** 2024-04-02

**Authors:** Kathrin Skoczynski, Andre Kraus, Christoph Daniel, Maike Büttner-Herold, Kerstin Amann, Mario Schiffer, Kristina Hermann, Leonie Herrnberger-Eimer, Ernst R. Tamm, Bjoern Buchholz

**Affiliations:** 1 Department of Nephrology and Hypertension, Friedrich-Alexander University Erlangen-Nürnberg, Erlangen, Germany; 2 Department of Nephropathology, Friedrich-Alexander University Erlangen-Nürnberg, Erlangen, Germany; 3 Institute of Human Anatomy and Embryology, University of Regensburg, Regensburg, Germany

**Keywords:** Fibronectin, Alpha 8 integrin, Kidney development, Branching, Nephron number

## Abstract

Complex interactions of the branching ureteric bud (UB) and surrounding mesenchymal cells during metanephric kidney development determine the final number of nephrons. Impaired nephron endowment predisposes to arterial hypertension and chronic kidney disease. In the kidney, extracellular matrix (ECM) proteins are usually regarded as acellular scaffolds or as the common histological end-point of chronic kidney diseases. Since only little is known about their physiological role in kidney development, we aimed for analyzing the expression and role of fibronectin. In mouse, fibronectin was expressed during all stages of kidney development with significant changes over time. At embryonic day (E) 12.5 and E13.5, fibronectin lined the UB epithelium, which became less pronounced at E16.5 and then switched to a glomerular expression in the postnatal and adult kidneys. Similar results were obtained in human kidneys. Deletion of fibronectin at E13.5 in cultured metanephric mouse kidneys resulted in reduced kidney sizes and impaired glomerulogenesis following reduced cell proliferation and branching of the UB epithelium. Fibronectin colocalized with alpha 8 integrin and fibronectin loss caused a reduction in alpha 8 integrin expression, release of glial-derived neurotrophic factor and expression of Wnt11, both of which are promoters of UB branching. In conclusion, the ECM protein fibronectin acts as a regulator of kidney development and is a determinant of the final nephron number.

**Supplementary Information:**

The online version contains supplementary material available at 10.1007/s00424-024-02954-9.

## Introduction

Embryonic kidney development is a complex process, which may explain why urinary tract malformations are among the most common birth defects in humans [2, 27]. In humans, the development of the final kidney, the metanephros, starts at embryonic day (E) 35 with reciprocal signaling between the Wolffian duct and the adjacent metanephric mesenchyme (MM) [32]. Nephrogenesis significantly depends on the formation of the ureteric bud (UB) and the nephron endowment at the branching UB tips [9]. In mouse, kidney development begins at E10.5 with the outgrowth of the UB from the Wolffian duct, which then invades the MM [9]. The UB, which later becomes the collecting duct system, begins to branch dichotomously and induces the MM to condense and undergo mesenchymal-to-epithelial transformation [20]. The condensed MM then forms renal vesicles, followed by comma- and s-shaped bodies, which later turn into the nephrons [20]. The inductive signaling is an essential step in kidney development as it induces UB outgrowth and subsequent UB branching. The glial-derived neurotrophic factor (GDNF) is produced by the MM cells and plays a key role in UB branching [29]. GDNF promotes UB cell proliferation and regulates the expression of the extracellular signaling molecule WNT11 in the UB tips, which in turn increases GDNF expression in a positive feedback loop [17]. GDNF deletion in mice results in significant defects in UB outgrowth and consequently renal agenesis [29].

Integrins are cell adhesion receptors which interact with several proteins of the extracellular matrix (ECM) and are involved in embryonic kidney development [22]. Integrins consist of the subunits α and β and are divided into three groups called laminin-, collagen-, and arginyl-glycyl-aspartic acid (RGD)-binding integrins [22]. The RGD-binding integrins are linked to several ECM ligands [22]. The RGD-binding integrin α8β1 (ITGA8) is expressed by MM cells and stimulation of ITGA8 significantly contributes to GDNF induction [24]. Similar to GDNF deletion, loss of ITGA8 in mouse leads to impaired UB outgrowth in the MM and results in renal agenesis [24, 29]. In addition, mutations of ITGA8 have been described as a genetic cause of bilateral renal agenesis in humans [15].

Next to the detrimental phenotypes provided by loss of GDNF or ITGA8, alterations in kidney development do not always have to result in renal agenesis but can affect the final kidney size and number of nephrons and glomeruli. In humans, nephron number between individuals varies from roughly 200,000 up to 2,000,000 nephrons, which exceeds the structural variability of most other organs [14]. Low nephron number increases the risk for chronic kidney disease (CKD) due to lower functional reserve and continuous hyperfiltration [6]. In addition, low nephron number also predisposes to arterial hypertension [5, 21]. Despite the significant impact of nephron endowment for health, the determinants of nephron number remain incompletely understood.

ECM proteins such as collagens, fibronectin, and laminins are often regarded as scaffolding proteins in the kidney providing structural stability or as an irrefutable histological sign of progressive CKD [7, 18]. However, ECM proteins are largely involved in signal transduction and therefore may affect kidney development as recently shown for the ECM protein nephronectin [3, 19]. Recently, fibronectin has been shown to mediate branching morphogenesis of mouse embryonic salivary glands and additionally was detected at early stages of mouse metanephric kidney development [28]. Fibronectin is a glycoprotein which exists as a dimer linked by a pair of disulfide bonds and exists in two types [26]. The soluble form which is secreted by cells and the insoluble species which occurs in fibrillar extracellular structures [26]. Fibronectin is ubiquitously expressed and involved in wound healing processes, cell adhesion, migration, growth, and differentiation [26].

Therefore, we investigated the expression of fibronectin during mouse and human kidney development in more detail and tested for its impact on nephrogenesis by deletion of fibronectin in ex vivo cultured metanephric mouse kidneys.

## Results

### Fibronectin is expressed during kidney development with significant changes of its localization over time

**Fig. 1:**
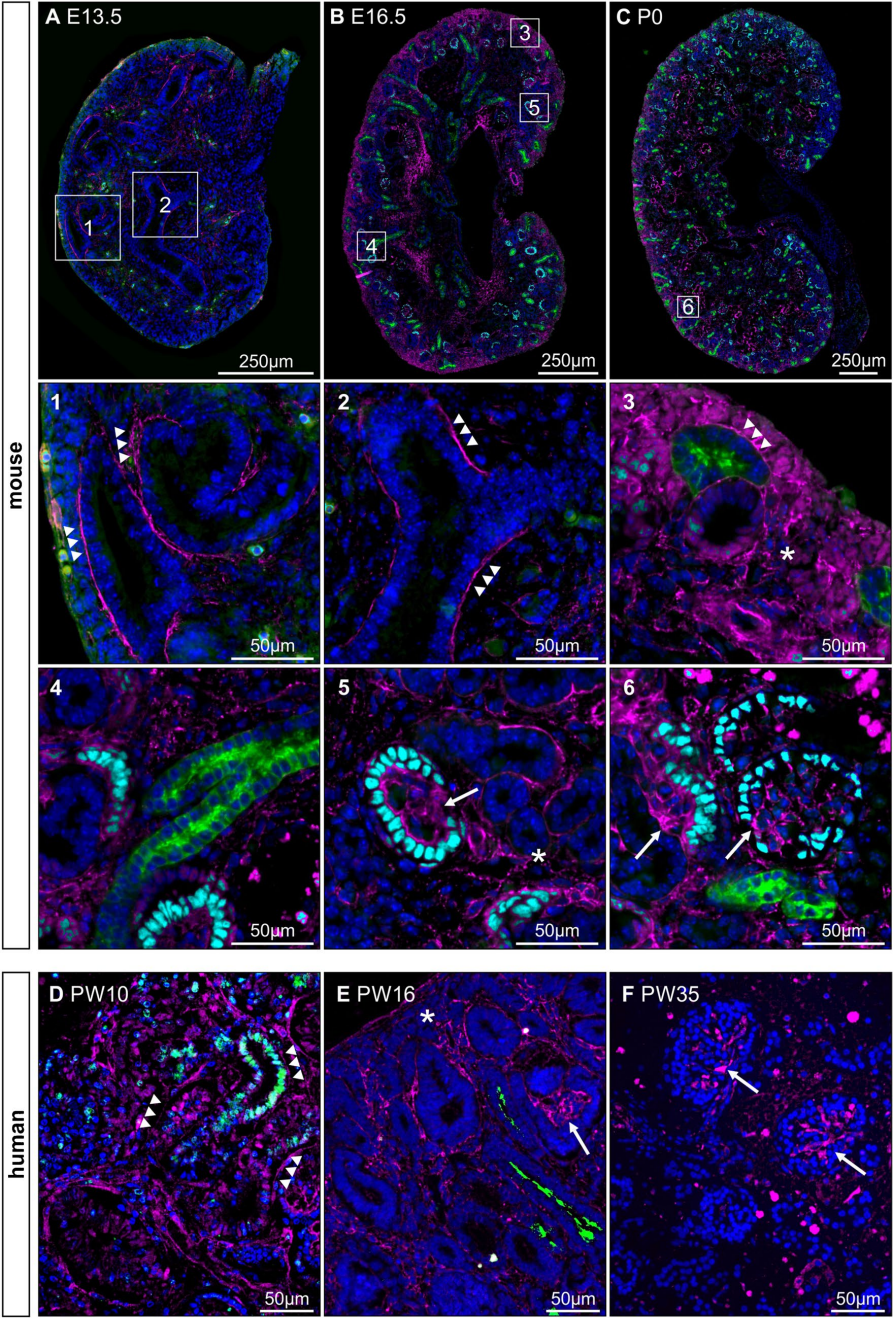
Expression and localization of fibronectin in kidney development. Fibronectin (magenta), dolichos biflorus agglutinin (DBA; green), as a marker for UB cells and collecting ducts, nuclei (DAPI; blue), and Wilms tumor protein (WT1; cyan) representing (pre-) glomerular structures (**A**–**C**) were stained at different stages of kidney development. **A**–**C** shows sections of mouse kidneys from embryonic day E13.5, E16.5 and postnatal day P0 which represent the change of fibronectin expression obtained from E12.5 until adulthood (extended in supplemental Fig. 1). Numbers (**1**–**6**) show magnifications indicated by squares in **A**–**C**. (**1**) Fibronectin lining ureteric bud epithelial cells (tips and trunks) indicated by arrowheads within the nephrogenic zone at E13.5 (and E12.5). (**2**) At the time from E12.5 to E13.5, fibronectin also lines ureteric bud cells that are not situated exclusively in the nephrogenic zone. (**3**) At E16.5, fibronectin can only rarely be found lining ureteric buds limited to the nephrogenic zone but becomes present in the primary interstitium (asterisk). (**4**) At E16.5, fibronectin is no longer detectable lining ureteric bud cells outside the nephrogenic zone. (**5**) At E16.5, fibronectin cannot only be detected within the primary interstitium (asterisk) but also becomes present within pre-glomerular structures (arrow). (**6**) At P0 (and adulthood), fibronectin is predominantly expressed in glomeruli. **D**–**F** show representative stainings of fetal human kidney sections at week 10 (**D**), 16 (**E**), and 35 (**F**) of pregnancy (PW). Arrowheads indicate fibronectin lining ureteric bud epithelial cells, asterisk marks interstitial fibronectin expression, and arrows indicate glomerular staining pattern

First, we characterized the expression of fibronectin in wild-type mouse kidneys using immunofluorescent stainings at different time points of kidney development. Metanephric kidneys were harvested at E12.5, E13.5, E16.5, P0, P7, and adulthood. Fibronectin expression was detected during all stages of nephrogenesis. However, the localization of fibronectin signal changed significantly over time. At E12.5 and E13.5, fibronectin primarily lined the UB branches and tips (Fig. 1A and Supplemental Fig. 1A, B) in the nephrogenic zone, but not limited to it, which corresponded to previous studies [10, 28]. In embryonic kidneys at E16.5, fibronectin was rarely detected around the ureteric bud epithelium limited to the nephrogenic zone. Instead, fibronectin was found in the primary interstitium, which was extended by subtle expression within the (pre-) glomerular structures (Fig. 1B and Supplemental Fig. 1C). At P0 and P7, fibronectin expression disappeared from the tubulo-interstitium and changed to a glomerular staining pattern (Fig. 1C and Supplemental Fig. 1D, E). In healthy adult kidneys, fibronectin appeared almost exclusively in the glomerular matrix (Supplemental Fig. 1F). Similar fibronectin expression patterns were found in human fetal kidneys at week 10, 16, 21, and 35 of pregnancy using immunofluorescence staining (Fig. 1D–F and Supplemental Fig. 1G-J). In all those stages, fibronectin expression was detected. Again, the pattern of fibronectin changed from interstitial localization lining the tubular structures at week 10 of pregnancy (Fig. 1D and Supplemental Fig. 1G) to a more diffuse interstitial expression at week 16 of pregnancy (Fig. 1E and Supplemental Fig. 1H). At week 21 and 35 of pregnancy, fibronectin staining was predominantly present within glomerular structures (Fig. 1F and Supplemental Fig. 1I, J). These findings reveal that fibronectin is expressed during kidney development of mouse and human. The striking localization along the UB branches suggests that fibronectin may be involved in UB branching morphogenesis of developing kidneys.

### *Tamoxifen-inducible deletion of fibronectin in *ex vivo* cultured metanephric kidneys*

**Fig. 2:**
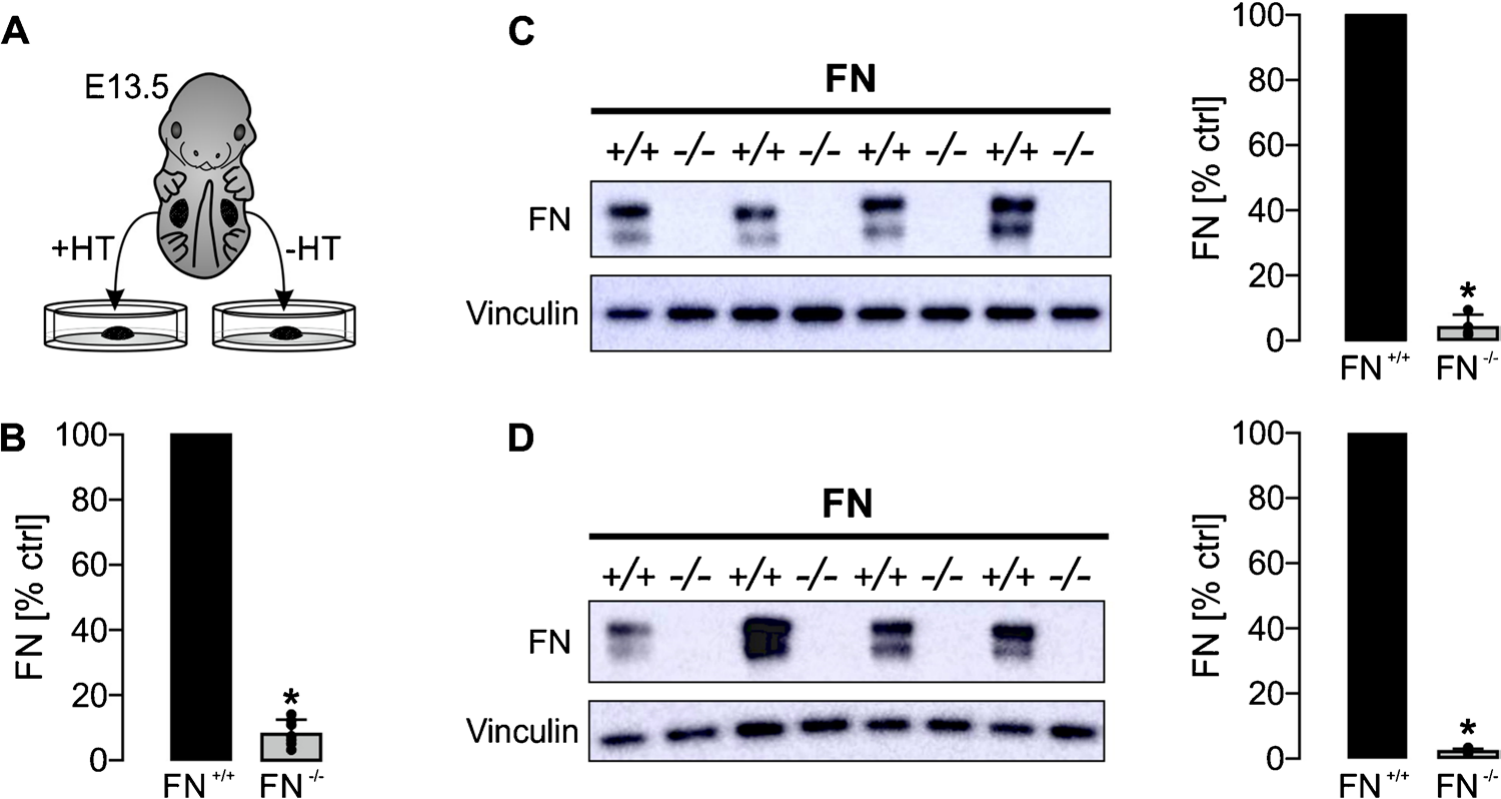
Fibronectin deletion in ex vivo cultured metanephric mouse kidneys. E13.5 metanephric kidney pairs (*n* = 11) were cultured ex vivo for 5 days. (Z)-4-hydroxytamoxifen (HT) was applied and the contralateral kidneys were maintained under control condition (illustrated in **A**). Application of HT resulted in a significant loss of fibronectin (FN^−/−^) 4 days later (day 5 of experiments) as indicated by real-time PCR (**B**) and western blot analysis (**C**). **C** Left shows representative western blot; right shows statistical analysis of fibronectin expression normalized for vinculin with FN^+/+^ set = 100%. + / + represents control kidneys (not induced with HT); − / − represents contralateral kidneys that were induced with HT to delete fibronectin. **D** Significant reduction of fibronectin was already noticed 48 h after application of HT by western blot analysis (*n* = 5 kidney pairs). *Significant compared to FN^+/+^

Constitutive knockout of fibronectin leads to embryonic death in mice due to defects in mesoderm, neural tube, and vascular development [13]. In order to study the role of fibronectin (FN) during embryonic kidney development, CAGG-Cre-ER™;FN^fl/fl^ mice were generated allowing tamoxifen-inducible deletion of fibronectin. Therefore, metanephric kidneys were dissected at E13.5 and cultured ex vivo on organotypic Millicell organotypic cell culture inserts for 5 days. One kidney of the kidney pairs was genetically deleted for fibronectin by application of (Z)-4-hydroxytamoxifen (HT) (500 nM) at day 1, whereas the contralateral kidney served as control (Fig. 2A). Real-time PCR showed significant reduction of fibronectin mRNA level 5 days after application of HT compared to the contralateral control kidneys (Fig. 2B). Additional western blot analysis confirmed these findings on protein level (Fig. 2C and Supplemental Fig. 2). Notably, significant reduction of fibronectin was already visible 48 h after application of HT in kidney culture indicating a short half-life in kidney development (Fig. 2D and Supplemental Fig. 2).

### Deletion of fibronectin resulted in reduced kidney sizes, impaired branching, and lower number of glomeruli

**Fig. 3:**
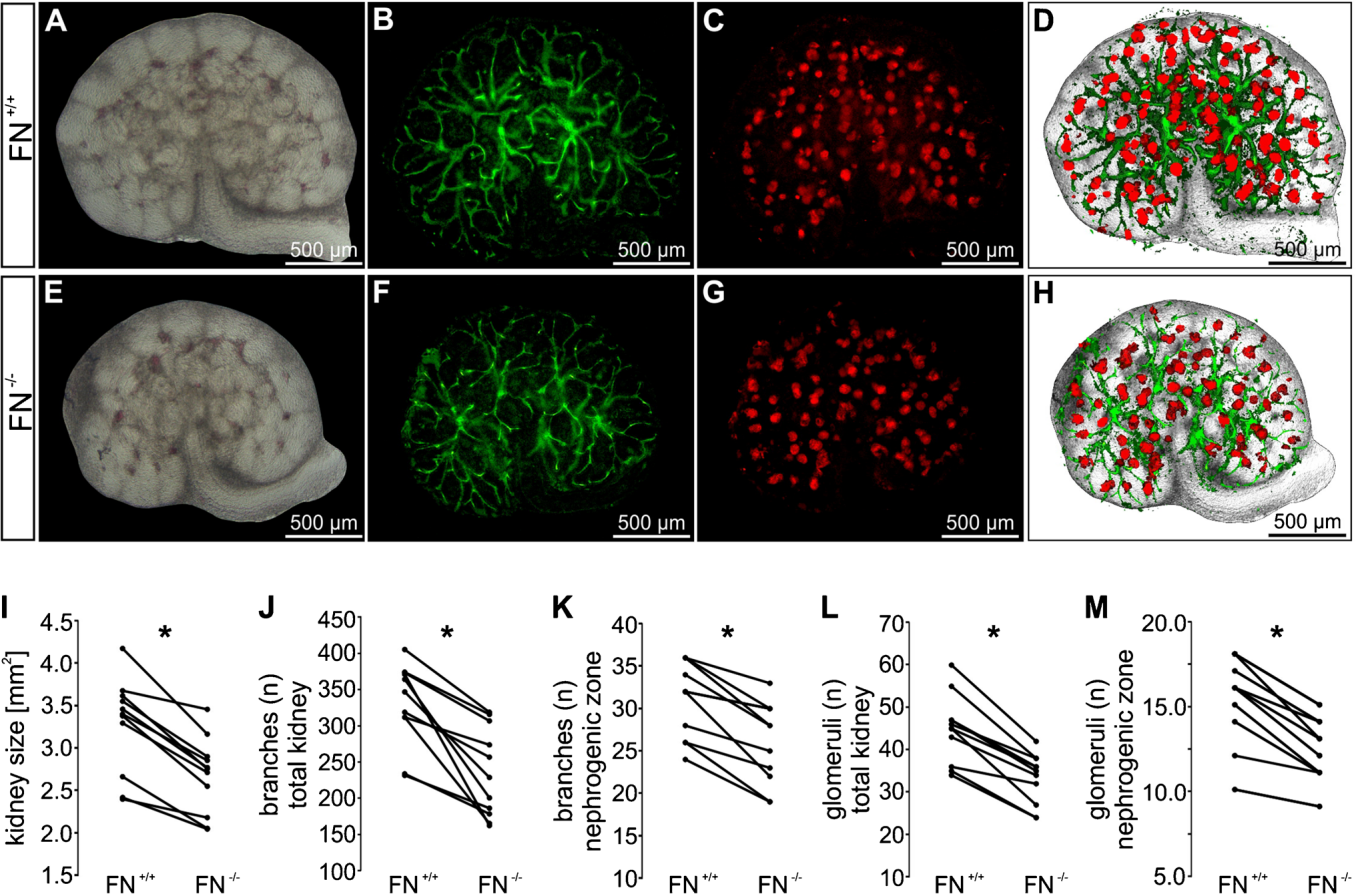
Loss of fibronectin resulted in reduced kidney size, impaired UB branching, and lower number of glomeruli. E13.5 metanephric kidney pairs (*n* = 11) were cultured ex vivo for 5 days. Deletion of fibronectin was induced at day 1 (FN^−/−^) and compared with contralateral kidneys cultured under control conditions (FN^+/+^) after 5 days of culture. Thereafter, kidney size, UB branches, and glomeruli were visualized by the use of extended depth of field images of whole mount kidneys in combination with bright-field microscopy (**A**, **E**), DBA staining (green) (**B**, **F**), and staining for Wilms tumor protein (WT1; red) (**C**, **G**), respectively. **D** and **H** show segmented images of the whole mount kidneys of **A**–**C** and **E**–**G**. **I**–**M** show quantification of kidney sizes (**I**), number of branches within the total kidney (**J**), number of branches within the nephrogenic zone (**K**), number of glomeruli within the total kidney (**L**), and number of glomeruli within the nephrogenic zone (**M**). *Significant compared to FN^+/+^

Metanephric kidney pairs were dissected at E13.5 and cultured ex vivo for 5 days. One kidney was treated with HT to induce fibronectin deletion (FN^−/−^) whereas the contralateral kidney served as fibronectin-competent control (FN^+/+^). To test for the role of fibronectin in nephrogenesis, whole mount kidneys were stained for UB branches and glomeruli and photographed at the end of day 5. Fibronectin-deleted kidneys were significantly smaller than control kidneys (Fig. 3A, E, I). Furthermore, UB branching morphogenesis was significantly impaired upon deletion of fibronectin (Fig. 3B, F, J). UB branching was particularly reduced within the nephrogenic zone (Fig. 3K). In addition, loss of fibronectin resulted in a reduced number of glomeruli (Fig. 3C, G, L) with a significant reduction of glomeruli within the nephrogenic zone (Fig. 3M).

**Fig. 4:**
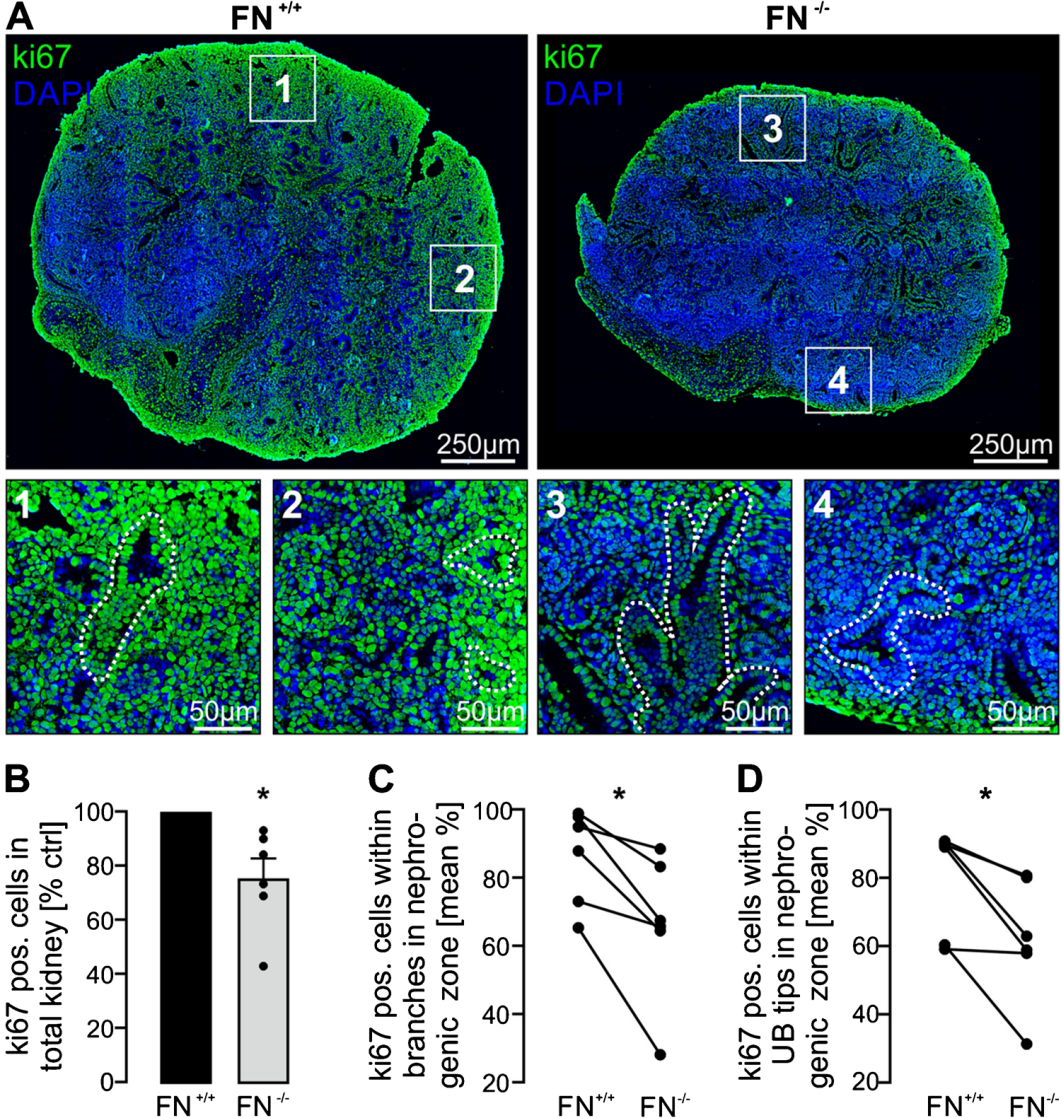
Loss of fibronectin resulted in reduced epithelial cell proliferation. Metanephric mouse kidneys (*n* = 6 kidney pairs) were harvested at E13.5 and cultured ex vivo ± application of HT for 5 days. **A** Kidney sections were stained for the proliferation marker ki67 (green signals) and nuclei (DAPI; blue). Upper row shows whole kidney sections; squares indicate magnifications depicted below with the corresponding numbers. (**1**–**4**) The white dotted lines illustrate representative UB branches localized within the nephrogenic zone of FN^+/+^ (**1**, **2**) and FN^−/−^ (**3**, **4**) kidneys. **B** Quantification of ki67-positive cells in relation to total kidney tissue area and normalized to FN^+/+^. **C** Quantification of ki67-positive cells within UB branches (tips and trunks) in the nephrogenic zone and normalized to the total number of UB branching cells. **D** Quantification of ki67-positive cells within UB tips in the nephrogenic zone and normalized to the total number of cells within the UB tips. *Significant compared to FN^+/+^

UB branching depends on cell proliferation [1, 23]. Therefore, kidneys were stained for the proliferation marker ki67 5 days after induction of fibronectin deletion. Ki67 signals were significantly reduced after deletion of fibronectin and normalized to the whole kidney tissue (Fig. 4A, B). The effect was largely driven by a reduction of ki67-positive cells within the UB branches in the nephrogenic zone shown by selective analyses of epithelial cells within UB branches and normalized to the total number of UB cells (Fig. 4A, C). A significant, reduction in cell proliferation was additionally found analyzing exclusively the UB tips in the nephrogenic zone (Fig. 4D). Similar results were obtained by the use of the proliferation marker PCNA (Supplemental Fig. 3). These findings indicate that fibronectin is necessary to induce proliferation of UB branching cells which consequently affects kidney size and nephron endowment.

### Fibronectin colocalizes with ITGA8 in metanephric kidneys and affects ITGA8-dependent signaling

**Fig. 5:**
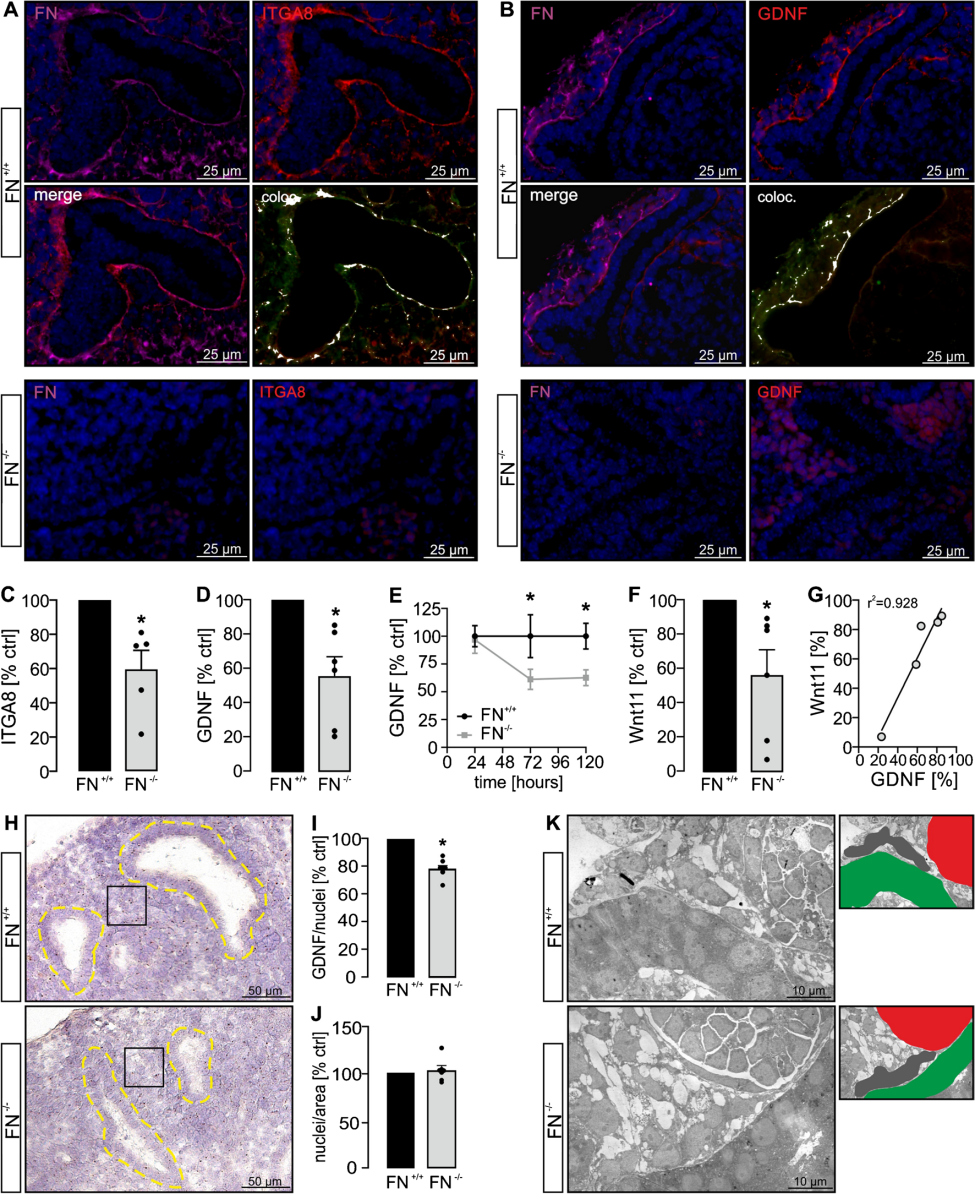
Fibronectin affects ITGA8-dependent signaling. Metanephric mouse kidneys (*n* = 5 kidney pairs) were harvested at E13.5 and cultured ex vivo ± HT for 5 days. **A** Fibronectin-competent kidneys (FN^+/+^) were stained for fibronectin (FN; magenta), ITGA8 (red), and nuclei (DAPI; blue). Both, fibronectin and ITGA8 staining lined the UB branches and showed significant colocalization (white signals). No fibronectin, but also no significant ITGA8 signal could be detected in the nephrogenic zone of FN-deficient kidneys (FN^−/−^). **B** Fibronectin-competent kidneys (FN^+/+^) were stained for fibronectin (FN; magenta), GDNF (red), and nuclei (DAPI; blue). Fibronectin and GDNF stainings lined the UB branches and were significantly colocalized (white signals). No fibronectin, but also no significant GDNF signal could be detected in FN-deficient kidneys (FN^−/−^). **C** ITGA8 mRNA level was significantly reduced in fibronectin-deleted kidneys (FN^−/−^) (FN^+/+^ set = 100%; *n* = 5 kidney pairs). **D** GDNF mRNA level was significantly reduced in fibronectin-deficient kidneys (FN^−/−^) (FN^+/+^ set = 100%; *n* = 6 kidney pairs). **E** ELISA assays performed after 24 h, 72 h, and 120 h of kidney culture revealed reduced concentration of GDNF in the medium of FN^−/−^ kidneys (FN^+/+^ set = 100%; *n* = 6 kidney pairs). **F** Wnt11 mRNA expression was significantly reduced in FN^−/−^ kidneys (FN^+/+^ set = 100%; *n* = 6 kidney pairs). **G** Correlation of GDNF and Wnt11 mRNA expression (*n* = 5 identical FN^−/−^ kidneys in **D** and **F**). **H** In situ hybridization of GDNF (*n* = 7 kidney pairs) was performed. Regions of interest (ROIs) covering the interstitial space between UB branches within the nephrogenic zone of each kidney were analyzed for GDNF signals and the number of nuclei. The square (black line) illustrates one exemplary ROI. Yellow dotted lines indicate UB branches. **I** FN^−/−^ kidneys showed a significantly lower expression of GDNF normalized to cell number. Each dot represents the mean value of all ROIs of one kidney. **J** The cell number (number of nuclei) was identical in FN^+/+^ and FN^−/−^ kidneys. Each dot represents the mean value of all ROIs of one kidney. **K** Electron microscopy images revealed a similar appearance of mesenchymal cells in FN^+/+^ and FN^−/−^ kidneys surrounding UB epithelial cells. Small photos on the right illustrate the structures displayed in the EM photo on the left. Gray: metanephric mesenchymal cells; green: UB epithelial branch; red: glomerular structure. *Significant compared to FN^+/+^

ITGA8 is involved in kidney development by promoting GDNF-mediated UB cell proliferation [4, 24]. Recently, the ECM protein nephronectin has been discovered as an important ligand to ITGA8 in kidney development [19]. Fibronectin has also been suggested as a ligand to ITGA8, however with a much lower affinity [30]. We next performed colocalization studies of fibronectin and ITGA8 and found significant colocalization of fibronectin and ITGA8 along the UB branches in metanephric mouse kidneys harvested at E13.5 predominantly in the nephrogenic zone (Fig. 5A and Supplemental Fig. 4A). Fibronectin deletion resulted in a significant reduction of ITGA8 signals in the nephrogenic zone (Fig. 5A and Supplemental Fig. 4B). Since ITGA8 stimulation results in GDNF release, we next stained for GDNF and again found significant colocalization of fibronectin and GDNF in E13.5 metanephric mouse kidneys (Fig. 5B and Supplemental Fig. 5A). Loss of fibronectin led to a strong reduction of GDNF signals (Fig. 5B and Supplemental Fig. 5B). In line with the reduction of ITGA8 staining pattern around the UB branches in the nephrogenic zone, ITGA8 mRNA was significantly reduced in whole mount kidney specimens after deletion of fibronectin (Fig. 5C). In addition, fibronectin deletion also resulted in a significantly reduced expression of GDNF mRNA (Fig. 5D). Next, we used ELISA assays to test for GDNF release into the medium over time after induction of fibronectin deletion compared to control kidneys. In line with the previous findings, loss of fibronectin led to a significantly reduced release of GDNF into the medium (Fig. 5E). Furthermore, deletion of fibronectin also resulted in significantly reduced mRNA levels of Wnt11 (Fig. 5F) which correlated with GDNF expression (Fig. 5G).

Next, we wondered if the reduction of GDNF (and subsequently Wnt11) may simply be explained by the structural loss of metanephric mesenchymal cells surrounding the UB epithelium in the nephrogenic zone. Therefore, we additionally performed in situ hybridization for GDNF (Fig. 5H), which on the one hand confirmed significant reduction of GDNF within the interstitial space of the nephrogenic zone in fibronectin-deleted kidneys (Fig. 5I) and on the other hand showed that the cell number within the analyzed areas was identical in fibronectin-competent and fibronectin-deficient kidneys (Fig. 5J). Furthermore, ultrastructural analyses by the use of electron microscopy revealed similar appearance of the interstitial cells surrounding the UB epithelial cells in the nephrogenic zone (Fig. 5K). These data rather support a lack of ITGA8-dependent signaling than structural defects or loss of MM cells upon deletion of fibronectin.

In summary, our data suggest that during kidney development fibronectin stimulates ITGA8 in MM cells which leads to activation of GDNF. GDNF then promotes UB cell proliferation and release of Wnt11.

## Discussion

Our study shows that the ECM protein fibronectin is expressed during kidney development and promotes UB branching. Therefore, fibronectin qualifies as a determinant of the final number of nephrons and glomeruli in the mature kidney.

Fibronectin is an ubiquitously expressed protein and involved in cell adhesion, growth, migration, and differentiation [26]. Two types of fibronectin are present in vertebrates: soluble fibronectin, which is produced in the liver and representing a major protein component of blood plasma, and insoluble cellular fibronectin, which is a major component of the ECM [26]. The latter is secreted by various cells as a soluble protein dimer and later on is assembled into an insoluble matrix [12]. Since we analyzed the kidneys ex vivo and therefore isolated from blood flow, the observed phenotypes were clearly driven by endogenous fibronectin. Although the ex vivo culture of metanephric kidneys is artificial, it provides additional advantages: (i) it allows to overcome the issue with embryonic lethality of the constitutive knockout and (ii) it allows direct comparison of the kidney pairs.

The strong fibronectin signal lining the UB branches suggests the UB branching epithelial cells as the source of fibronectin with it being secreted from the UB cells as discussed by other groups before [28, 35]. However, we cannot exclude that other cells than UB cells or other cells in addition to UB cells may secrete fibronectin in metanephric kidneys. A similar expression pattern of fibronectin was observed in fetal human kidneys. The human sample sizes were small and we cannot rule out that unknown underlying genetic defects or other alterations, which led to the spontaneous abortions may have affected fibronectin expression in these kidneys. However, given the high analogy to the mouse samples, the findings indicate that the expression patterns may be representative.

Recently, the ECM protein nephronectin has been shown to promote UB branching by stimulation of ITGA8 and downstream-mediated release of GDNF [19]. Knockout of nephronectin frequently resulted in renal agenesis [19] whereas in our study deletion of fibronectin, which likely acts on the same signaling pathway, resulted in smaller kidneys with apparently intact architecture but reduced nephrons. There are several explanations for this discrepancy: Linton et al. used mice with a complete knockout of nephronectin [19], which therefore may affect kidney development much earlier than in our model where we deleted fibronectin at E13.5. Induced deletion was necessary since complete lack of fibronectin leads to embryonic death in mouse before E10.5 [13]. Therefore, loss of fibronectin at an earlier stage may be more deleterious. In addition, induced deletion means that the protein has been generated until the time point of intervention and the following effects depend on the half-life of the protein. However, we show that already 48 h after induction, fibronectin expression was largely reduced. Affinity of a ligand to its receptor is of course also noteworthy. Although fibronectin and nephronectin are both ligands of ITGA8, the affinity of fibronectin to ITGA8 is approximately 100-fold lower than that of nephronectin [30]. This could mean that fibronectin may rather act as a modulator or fine tuner of ITGA8-dependent signaling. The latter idea would fit to a model where fibronectin may be a factor contributing to the determination of the final nephron number and not necessarily resulting in renal agenesis if its function is altered. The significant reduction of ITGA8 staining patterns around the UB branches in the nephrogenic zone upon deletion of fibronectin further supports the idea that fibronectin acts via ITGA8 signaling pathways. ITGA8 has been shown to be expressed by nephron progenitor cells in the nephrogenic zone [16]. The underlying mechanism that leads to reduced expression of ITGA8 upon loss of fibronectin in this specific compartment remains unclear. Our data suggest that it is not due to a simple loss of mesenchymal cells as the number of interstitial cells was identical in fibronectin-competent and -deficient kidneys. Therefore, progenitor cells could either miss the signal that induces expression of ITGA8 or they fail to be recruited into the compartment and get replaced by other cells. In either case, GDNF signaling would be impaired and affect UB cell proliferation as shown in our experiments. However, we cannot exclude that additional signaling pathways may be involved.

Mutations in fibronectin have been linked to a particular renal disease, called glomerulopathy with fibronectin deposits (GFND, OMIM: 601894). GFND is a rare autosomal dominant disease characterized by proteinuria, microscopic hematuria, hypertension, and massive fibronectin deposits in the mesangium and subendothelial space, subsequently leading to end-stage renal failure [33]. Recently, mutations localized in the integrin-binding domain in GFND patients have been detected [25]. Although the disease is primarily regarded as being caused by massive fibronectin deposits, it would be interesting (and challenging) to analyze if these patients also suffer from reduced nephron mass.

Low nephron number predisposes to arterial hypertension [5, 21]. Recently, in a genome-wide association study, a variant of fibronectin was linked to arterial hypertension [34]. The underlying mechanism remained elusive. It is intriguing to speculate if this variant might impair kidney development and by this predispose to arterial hypertension. Given the ubiquitous expression and function of fibronectin, there are of course many other potential explanations for this observation.

In kidney research, ECM proteins are often regarded as acellular scaffolds that provide structural stability or as the unfortunate common histological end-point of progressive, chronic kidney diseases [7]. Here, we show that the ECM protein fibronectin is significantly involved in metanephric kidney development affecting ITGA8 receptor-dependent signaling. Therefore, fibronectin variants with reduced or lacking binding affinity to ITGA8 may lead to lower nephron number and predispose to arterial hypertension and CKD. The latter is speculative at this point but may deserve attention in future research projects.

## Methods

### Human fetal kidneys

Formalin-fixed paraffin embedded sections of human fetal kidneys of week 10, 16, 21, and 35 of pregnancy were analyzed with permission provided by the local Ethics committee (reference number 4415). The causes for intrauterine death or abortion can only partially be provided. Three out of four cases were spontaneous abortions of unclear origin. In the fetus of week 35 of pregnancy, fetal death occurred due to hemorrhagic shock of the mother with subsequent severe fetal anemia.

### Animals

Animal experiments were approved by the local institutional review board and all animal experiments complied with the United Kingdom Animals Act, 1986, and associated guidelines, EU Directive 2010/63/EU for animal experiments. Experiments were approved by the local Ethics Committee of the Government of Unterfranken/Wuerzburg (TS-2/2022 MedIV). Mice carrying the loxP-flanked conditional alleles of fibronectin (FN^fl/fl^) were kindly provided by Prof. Reinhard Fässler (Max Planck Institute of Biochemistry, Martinsried, Germany). Mice were crossed with CAGG-Cre-ER™ mice on a C57BL/6 background from Jacksons Laboratory that express Cre-recombinase after tamoxifen treatment in order to receive homozygous CAGG-Cre-ER™;FN^fl/fl^ mice. Cre-negative littermates were used for the fibronectin expression characterization of wild-type mice and non-tamoxifen induced mice served as control in the embryonic kidney culture experiments.

### Immunohistochemistry and antibodies

Whole mount kidneys were stained with FITC-conjugated dolichos biflorus agglutinin (DBA; 1:500, Vector Laboratories, USA) and polyclonal rabbit anti-Wilms tumor protein (WT1; 1:500, abcam, Berlin, Germany) after 5 days of ex vivo culture. As secondary antibody anti-rabbit IgG AlexaFluor® antibody (1:1000; Thermo Fisher Scientific, Inc., Erlangen, Germany) was used. For analyses of fibronectin expression and localization at different time points during kidney development, mouse kidneys from E12.5, E13.5, E16.5, P0, P7, and adulthood comprising *n* = 5–8 kidneys per time point and human kidneys from week 10, 16, 21, and 35 of pregnancy were used. Two-micron thick kidney sections were stained with polyclonal rabbit anti-fibronectin (1:2000, Dako, Denmark). Glomerular structures were stained with monoclonal rabbit anti-Wilms tumor protein (WT-1) (1:500, abcam, Berlin). Binding of the primary WT-1 antibody was visualized with secondary donkey anti-rabbit antibody conjugated with AlexaFluor® 555 (1:1000, Molecular Probes, Invitrogen) and fibronectin was visualized by the use of a secondary donkey anti-rabbit antibody conjugated with AlexaFluor® 647 (1:1000, Molecular Probes, Invitrogen). UB branches and the collecting ducts were stained with FITC-conjugated DBA (1:500, Vector Laboratories, USA) and nuclei were visualized by the use of DAPI. Ki67 staining was performed using monoclonal rabbit anti-Ki67 antibody (1:100, Linaris, Dossenheim, Germany) and signals were amplified by the use of the Vectastain Elite ABC Kit (Vector Laboratories, Burlingame, CA) according to the manufacturer’s instructions. PCNA was stained by the use of mouse monoclonal anti-PCNA antibody (1:50; Dako) followed by the use of an ABC M.O.M. kit (Vector). ITGA8 was stained by the use of polyclonal goat anti-integrin alpha 8 antibody (1:50; R&D Systems, Minneapolis) and GDNF was stained by the use of polyclonal rabbit anti-GDNF antibody (1:100; Bioss antibodies, USA). Signals were visualized using secondary donkey anti-goat conjugated with AlexaFluor® 555 and secondary donkey anti-rabbit antibody conjugated with AlexaFluor® 647 (both 1:1000, Molecular Probes, Invitrogen). Signals were analyzed with a DM6000B fluorescence microscope (Leica, Wetzlar, Germany), and photographs were taken with a Leica DFC 450C camera. Significance of co-localization was analyzed and visualized by the use of ImageJ (V.1.45) and the colocalization finder algorithm by Christophe Laummonerie and Jerome Mutterer (Institut de Biologie Moleculaire des Plantes, Strasbourg, France) as described previously [11]. For quantification of ki67 and PCNA, the color deconvolution algorithm (ImageJ) was applied to dissect the different signals from *n* = 6 kidney pairs, followed by binarization and particle analysis to obtain the ratio of the number of all positive cells within the total kidney tissue area (normalized to mm^2^ tissue). In addition, all DBA-positive branches localized in the nephrogenic zone were dissected by marking them as regions of interest by the use of ImageJ. All ki67 or PCNA-positive cells within the branches then were quantified and set in ratio to the total cell number of the branches. In summary, a total number of *n* = 145 branches from *n* = 6 individual kidney pairs was analyzed. In addition, positive cells exclusively localized within the tips of the UB branches in the nephrogenic zone were analyzed accordingly. ITGA8, GDNF, and WNT11 signals were quantified as described previously [8]. Briefly, fluorescent signals from *n* = 5–6 individual kidney pairs were turned into 8-bit images after subtracting background (ImageJ) and a predefined threshold was used for all images to capture the signals, which then were set in ratio to the whole tissue area. All analyses were performed in a blinded manner.

### Embryonic kidney culture and morphometric analysis

Metanephric kidneys were harvested from embryonic CAGG-Cre-ER™;FN^fl/fl^ mice at embryonic day E13.5. Therefore, embryonic kidney pairs from *n* = 11 individual mice were cultured ex vivo on transparent Millicell organotypic cell culture inserts (Merck Millipore, Billerica, MA, USA) and maintained in a 37 °C humidified CO_2_ incubator for 5 days. For fibronectin deletion (FN^−/−^), one metanephric kidney was treated with (Z)-4-hydroxytamoxifen (HT; 500 nM, Sigma-Aldrich) whereas the contralateral kidney was treated with control medium only and served as control (FN^+/+^). HT was diluted in DMEM culture medium, containing 2 mM L-glutamine, 10 mM HEPES, 10 mM insulin, 5.5 µg/ml transferrin, 6.7 ng/ml sodium selenite, 32 pg/ml triiodothyronine, 250 U/ml penicillin, 250 µg/ml streptomycin, and 25 ng/ml prostaglandin E, and added below the culture inserts. Medium was changed after 24 h and 72 h. After 5 days, whole kidneys were fixed in paraformaldehyde (4%) and stained with FITC-conjugated dolichos biflorus agglutinin (DBA) to illustrate branching morphogenesis and for Wilms tumor protein (WT1) to depict glomerulogenesis. Metanephric kidneys stained with FITC-DBA and for WT1 were photographed along the z-axis providing a series of 20 photos covering the kidney from top to bottom. Obtained photos then were combined into a single extended depth of field image by the use of the “full focus” algorithm of the BZ-9000 analyzer software (V.2.1., Keyence, Japan). The resulting micrograph then was further processed by the use of ImageJ (NIH, V.1.45) comprising “subtraction of background,” “contrast enhancement,” “filter mean” providing a blurred image and thereby reducing background noise, “conversion into 8-bit grayscale image,” “threshold,” “removal of outliers” which in some cases was extended by “manual excision” of outliers or artifacts always including the ureter. The resulting photo then was converted into a skeletal representation of the branching architecture by the use of “skeletonize” which then provided the number of branches by the use of the “analyze skeleton” algorithm. Since WT1 does not only stain for glomeruli but also for the cap mesenchyme, an additional algorithm was applied for the count of glomeruli by defining “circularity” values followed by the “particle analysis” algorithm that separates glomeruli from other (non-circular) structures as described previously [31]. In addition, bright field depth of field images were captured along the z-axis to obtain kidney sizes. Kidney sizes, number of branches, and glomeruli were compared to the contralateral control kidney.

### Western blotting

Proteins were isolated from embryonic mouse kidneys using a sample buffer containing 50 mM Tris–HCl, 150 mM NaCl, 10 mM EDTA, 1% sodium deoxycholate, 0.1% SDS, 1% protease inhibitor mixture (Roche, cOmplete, EDTA-free, Mannheim, Germany), and 1% Triton X-100. Proteins were separated using NuPAGE 3–8% Tris–Acetate Protein Gels (Life Technologies/Gibco®, Karlsruhe, Germany). For the detection of fibronectin, proteins were blotted using an iBlot 2 Dry Blotting System (Thermo Fisher Scientific, Inc., Erlangen, Germany) to a polyvinylidene difluoride membrane (GE Healthcare Europe GmbH, Munich, Germany). Membrane was then incubated with primary anti-fibronectin (1:500; Dako, Santa Clara, USA) overnight. Proteins were visualized using horseradish peroxidase-conjugated secondary antibody and ECL detection. Monoclonal anti-mouse vinculin (1:4000, Novus Biologicals, USA) was used as loading control.

### Real-time PCR

RNA from embryonic kidneys was extracted by the use of the peqGold Total RNA-Kit (VWR International GmbH, Darmstadt, Germany) according to the manufacturer’s instructions. SYBR-Green-based real-time PCR was performed using StepOnePlus (Applied Biosystems, Foster City, CA, USA). Messenger RNA (mRNA) expression levels were normalized to 18S using the ΔΔCt method. All primer sequences are listed in Supplemental Table 1.

### ELISA

For quantitative detection of mouse GDNF protein release into the medium, enzyme-linked immunosorbent assay (ELISA) was performed using the Mouse GDNF CLIA Kit (BIOZOL Diagnostica Vertrieb GmbH, Eching, Germany) according to the manufacturer’s instructions. For this analysis, metanephric kidneys were harvested from embryonic CAGG-Cre-ER™;FN^fl/fl^ at E13.5 and cultured ex vivo on culture inserts for 5 days (*n* = 6 kidney pairs). One of the kidneys was exposed to HT to induce fibronectin deletion (FN^−/−^) and the other kidney served as control (FN^+/+^). Medium was obtained after 24 h, 72 h, and 120 h and luminescence was measured using GloMax Microplate Reader (Promega GmbH, Walldorf).

### In situ hybridization

For detection of GDNF mRNA, in situ hybridization was performed using the RNAscope 2.5 HD Detection Brown Kit (ACD 322310, Advanced Cell Diagnostics ACD, Hayward, CA) and a Mm-Gdnf-C1 probe (ACD 1081821-C1), according to the manufacturer’s instructions. Tissue sections of 4 µm thickness were kept at 60 °C for 1 h, deparaffinized in xylene, dehydrated in ethanol, and blocked with peroxidase. Afterwards, the slides were boiled in antigen retrieval buffer at 95 °C for 15 min and digested with protease at 40 °C for 30 min. The target-specific probe was than hybridized with the slides in the HybEZ oven (ACD) at 40° for 2 h, followed by amplifications steps according to the manufacturer’s instructions. Signals were detected using 3,3′-diaminobenzidine and the slides were mounted with EcoMount mounting medium (EML897L, Biocare Medical). The slides were photographed with a Leica DM6000B microscope and a Leica DFC 450C camera. For quantification of GDNF signals, the interstitial space between UB branches in the nephrogenic zone was subdivided into uniform squares serving as regions of interests (ROIs) in order to obtain GDNF signals per ROI by the use of Image J. Then, the mean value of all ROIs of each kidney was determined. The same was done to obtain the number of nuclei within the analyzed ROIs. Signals from *n* = 7 kidney pairs (E13.5) were analyzed.

### Electron microscopy

Metanephric kidneys were fixed in glutaraldehyde (2.5%) and formalin (2.5%) buffered in 0.1 M PBS (pH 7.6). The embryonic kidneys were washed with PBS, treated with OsO_4_ (1%) for 60 min, and stained with Uranyless (1%) (Science services GmbH, Munich, Germany). After dehydration, metanephric kidneys were embedded in epoxy Araldite resin (Serva Electrophoresis GmbH, Heidelberg, Germany). Ultrathin Sects. (80 nm) were performed with an ultramicrotome (Leica, Wetzlar, Germany), transferred to Formvar-coated cooper grids, and rinsed in lead citrate buffer. Analysis was performed using a SIGMA scanning electron microscope with STEM detector at 28 kV (Zeiss, Oberkochen, Germany).

### Statistical analysis

Data are expressed as mean ± SEM. An unpaired *t*-test was applied to compare the differences between two groups; a paired *t*-test was used for matched observations (kidney pairs). Wilcoxon signed-rank test for columns statistics was used for relative values. *P* < 0.05 was considered statistically significant.

### Supplementary Information

Below is the link to the electronic supplementary material.

Supplementary file1 (PDF 20465 KB)

## Data Availability

Original data are available from the authors upon reasonable request.

## References

[1] Affolter M, Bellusci S, Itoh N, Shilo B, Thiery J-P, Werb Z (2003). Tube or not tube: remodeling epithelial tissues by branching morphogenesis. Dev Cell.

[2] Airik R, Kispert A (2007). Down the tube of obstructive nephropathies: the importance of tissue interactions during ureter development. Kidney Int.

[3] Alcorn D, Maric C, McCausland J (1999). Development of the renal interstitium. Pediatr Nephrol Berl Ger.

[4] Brandenberger R, Schmidt A, Linton J, Wang D, Backus C, Denda S, Müller U, Reichardt LF (2001). Identification and characterization of a novel extracellular matrix protein nephronectin that is associated with integrin alpha8beta1 in the embryonic kidney. J Cell Biol.

[5] Brenner BM, Garcia DL, Anderson S (1988). Glomeruli and blood pressure. Less of one, more the other?. Am J Hypertens.

[6] Buchholz B, Schley G, Eckardt KU (2016). The impact of hypoxia on nephrogenesis. Curr Opin Nephrol Hypertens.

[7] Bülow RD, Boor P (2019). Extracellular matrix in kidney fibrosis: more than just a scaffold. J Histochem Cytochem Off J Histochem Soc.

[8] Cabrita I, Kraus A, Scholz JK, Skoczynski K, Schreiber R, Kunzelmann K, Buchholz B (2020). Cyst growth in ADPKD is prevented by pharmacological and genetic inhibition of TMEM16A in vivo. Nat Commun.

[9] Costantini F, Kopan R (2010). Patterning a complex organ: branching morphogenesis and nephron segmentation in kidney development. Dev Cell.

[10] Ekblom P (1981). Formation of basement membranes in the embryonic kidney: an immunohistological study. J Cell Biol.

[11] Forschbach V, Goppelt-Struebe M, Kunzelmann K, Schreiber R, Piedagnel R, Kraus A, Eckardt KU, Buchholz B (2015). Anoctamin 6 is localized in the primary cilium of renal tubular cells and is involved in apoptosis-dependent cyst lumen formation. Cell Death Dis.

[12] Geiger B, Bershadsky A, Pankov R, Yamada KM (2001). Transmembrane crosstalk between the extracellular matrix–cytoskeleton crosstalk. Nat Rev Mol Cell Biol.

[13] George EL, Georges-Labouesse EN, Patel-King RS, Rayburn H, Hynes RO (1993). Defects in mesoderm, neural tube and vascular development in mouse embryos lacking fibronectin. Dev Camb Engl.

[14] Hughson M, Farris AB, Douglas-Denton R, Hoy WE, Bertram JF (2003). Glomerular number and size in autopsy kidneys: the relationship to birth weight. Kidney Int.

[15] Humbert C, Silbermann F, Morar B, Parisot M, Zarhrate M, Masson C, Tores F, Blanchet P, Perez M-J, Petrov Y, Khau Van Kien P, Roume J, Leroy B, Gribouval O, Kalaydjieva L, Heidet L, Salomon R, Antignac C, Benmerah A, Saunier S, Jeanpierre C (2014). Integrin alpha 8 recessive mutations are responsible for bilateral renal agenesis in humans. Am J Hum Genet.

[16] Ihermann-Hella A, Hirashima T, Kupari J, Kurtzeborn K, Li H, Kwon HN, Cebrian C, Soofi A, Dapkunas A, Miinalainen I, Dressler GR, Matsuda M, Kuure S (2018). Dynamic MAPK/ERK activity sustains nephron progenitors through niche regulation and primes precursors for differentiation. Stem Cell Rep.

[17] Kispert A, Vainio S, Shen L, Rowitch DH, McMahon AP (1996). Proteoglycans are required for maintenance of Wnt-11 expression in the ureter tips. Dev Camb Engl.

[18] Lausecker F, Lennon R, Randles MJ (2022). The kidney matrisome in health, aging, and disease. Kidney Int.

[19] Linton JM, Martin GR, Reichardt LF (2007). The ECM protein nephronectin promotes kidney development via integrin alpha8beta1-mediated stimulation of Gdnf expression. Dev Camb Engl.

[20] Little MH, McMahon AP (2012). Mammalian kidney development: principles, progress, and projections. Cold Spring Harb Perspect Biol.

[21] Luyckx VA, Bertram JF, Brenner BM (2013). Effect of fetal and child health on kidney development and long-term risk of hypertension and kidney disease. Lancet.

[22] Mathew S, Chen X, Pozzi A, Zent R (2012). Integrins in renal development. Pediatr Nephrol Berl Ger.

[23] Meyer TN, Schwesinger C, Bush KT, Stuart RO, Rose DW, Shah MM, Vaughn DA, Steer DL, Nigam SK (2004). Spatiotemporal regulation of morphogenetic molecules during in vitro branching of the isolated ureteric bud: toward a model of branching through budding in the developing kidney. Dev Biol.

[24] Müller U, Wang D, Denda S, Meneses JJ, Pedersen RA, Reichardt LF (1997). Integrin alpha8beta1 is critically important for epithelial-mesenchymal interactions during kidney morphogenesis. Cell.

[25] Ohtsubo H, Okada T, Nozu K, Takaoka Y, Shono A, Asanuma K, Zhang L, Nakanishi K, Taniguchi-Ikeda M, Kaito H, Iijima K, Nakamura S-I (2016). Identification of mutations in FN1 leading to glomerulopathy with fibronectin deposits. Pediatr Nephrol Berl Ger.

[26] Pankov R, Yamada KM (2002). Fibronectin at a glance. J Cell Sci.

[27] Prato AP, Musso M, Ceccherini I, Mattioli G, Giunta C, Ghiggeri GM, Jasonni V (2009). Hirschsprung disease and congenital anomalies of the kidney and urinary tract (CAKUT): a novel syndromic association. Medicine (Baltimore).

[28] Sakai T, Larsen M, Yamada KM (2003). Fibronectin requirement in branching morphogenesis. Nature.

[29] Sánchez MP, Silos-Santiago I, Frisén J, He B, Lira SA, Barbacid M (1996). Renal agenesis and the absence of enteric neurons in mice lacking GDNF. Nature.

[30] Sato Y, Uemura T, Morimitsu K, Sato-Nishiuchi R, Manabe R-I, Takagi J, Yamada M, Sekiguchi K (2009). Molecular basis of the recognition of nephronectin by integrin alpha8beta1. J Biol Chem.

[31] Schley G, Scholz H, Kraus A, Hackenbeck T, Klanke B, Willam C, Wiesener MS, Heinze E, Burzlaff N, Eckardt KU, Buchholz B (2015). Hypoxia inhibits nephrogenesis through paracrine Vegfa despite the ability to enhance tubulogenesis. Kidney Int.

[32] Shah MM, Sampogna RV, Sakurai H, Bush KT, Nigam SK (2004). Branching morphogenesis and kidney disease. Development.

[33] Strøm EH, Banfi G, Krapf R, Abt AB, Mazzucco G, Monga G, Gloor F, Neuweiler J, Riess R, Stosiek P (1995). Glomerulopathy associated with predominant fibronectin deposits: a newly recognized hereditary disease. Kidney Int.

[34] Warren HR, Evangelou E, Cabrera CP, Gao H, Ren M, Mifsud B, Ntalla I, Surendran P, Liu C, Cook JP, Kraja AT, Drenos F, Loh M, Verweij N, Marten J, Karaman I, Lepe MPS, O’Reilly PF, Knight J, Snieder H, Kato N, He J, Tai ES, Said MA, Porteous D, Alver M, Poulter N, Farrall M, Gansevoort RT, Padmanabhan S, Mägi R, Stanton A, Connell J, Bakker SJL, Metspalu A, Shields DC, Thom S, Brown M, Sever P, Esko T, Hayward C, van der Harst P, Saleheen D, Chowdhury R, Chambers JC, Chasman DI, Chakravarti A, Newton-Cheh C, Lindgren CM, Levy D, Kooner JS, Keavney B, Tomaszewski M, Samani NJ, Howson JMM, Tobin MD, Munroe PB, Ehret GB, Wain LV, International Consortium of Blood Pressure (ICBP) 1000G Analyses, BIOS Consortium, Lifelines Cohort Study, Understanding Society Scientific group, CHD Exome+ Consortium, ExomeBP Consortium, T2D-GENES Consortium, GoT2DGenes Consortium, Cohorts for Heart and Ageing Research in Genome Epidemiology (CHARGE) BP Exome Consortium, International Genomics of Blood Pressure (iGEN-BP) Consortium, UK Biobank CardioMetabolic Consortium BP working group (2017). Genome-wide association analysis identifies novel blood pressure loci and offers biological insights into cardiovascular risk. Nat Genet.

[35] Ye P, Habib SL, Ricono JM, Kim N-H, Choudhury GG, Barnes JL, Abboud HE, Arar MY (2004). Fibronectin induces ureteric bud cells branching and cellular cord and tubule formation. Kidney Int.

