# Supplementary material for: The extracellular matrix protein fibronectin promotes metanephric kidney development
Source: Pflugers Arch. 2024 Apr 2;476(6):963–74. doi: 10.1007/s00424-024-02954-9 (PMC11139724; doi:10.1007/s00424-024-02954-9)

Supplementary Information

Supplemental Table 1. PCR primer sequences.

**Gene**	**Forward primer (5’-3’)**	**Reverse primer (5’-3’)**
Fibronectin	AAGGTTCGGGAAGAGGTTGT	CCGTGTAAGGGTCAAAGCAT
ITGA8	TGACACCACCAACAACAGG	AGTTCTCCAGTGATACAAAGGG
GDNF	CGCTGACCAGTGACTCCAAT	CTGCCGCTTGTTTATCTGGT
Wnt11	CCAAGCCAATAAACTGATGCG	GCATTTACACTTCGTTTCCAGGG
Rn18S	TGATTAAGTCCCTGCCCTTTGTA	CGATCCGAGGGCCTCACTA

Supplemental Figure 1. Expression and localization of fibronectin in kidney development. Fibronectin (magenta), dolichos biflorus agglutinin (DBA; green), as a marker for UB cells and collecting ducts, and nuclei (DAPI; blue) were stained at different stages of kidney development. (**A-F**) show representative stainings of mouse kidneys at embryonic day E12.5 (**A**), E13.5 (**B**), E16.5 (**C**), postnatal day P0 (**D**), P7 (**E**), and adult kidney (**F**). (**G-J**) show representative stainings of fetal human kidney sections at week 10 (**G**), 16 (**H**), 21 (**I**) and 35 (**J**) of pregnancy (PW). Arrowheads indicate fibronectin lining ureteric bud epithelial cells, asterisks mark interstitial fibronectin expression, and arrows indicate glomerular staining pattern.

Supplemental Figure 2. Uncropped blots. Figure shows uncropped blots stained for FN and Vinculin from figure 2C and 2D.

Supplemental Figure 3. Loss of fibronectin resulted in reduced epithelial cell proliferation. Metanephric mouse kidneys (n=6 kidney pairs) were harvested at E13.5 and cultured *ex vivo* ± application of HT for five days. (**A**) Kidney sections were stained for the proliferation marker PCNA (brown signals). Upper row shows whole kidney sections, squares indicate magnifications depicted below with the corresponding numbers. (**1-4**) The black dotted lines illustrate representative UB branches localized within the nephrogenic zone of $FN^{+/+}$ (1,2) and $FN^{-/-}$ (3,4) kidneys. (**B**) Quantification of PCNA-positive cells in relation to total kidney

tissue area and normalized to $FN^{+/+}$. (**C**) Quantification of PCNA-positive cells within UB branches (tips and trunks) in the nephrogenic zone and normalized to the total number of UB branching cells. (**D**) Quantification of PCNA-positive cells within UB tips in the nephrogenic zone and normalized to the total number of cells within the UB tips. * significant compared to $FN^{+/+}$.

Supplemental Figure 4. Colocalization of fibronectin and ITGA8. Representative photos of metanephric mouse kidneys that were harvested at E13.5 and cultured *ex vivo* ± HT for five days. Thereafter, FN-competent kidneys ($FN^{+/+}$) and FN-deficient kidneys ($FN^{-/-}$) were stained for fibronectin (FN; magenta), ITGA8 (red), nuclei (DAPI; blue) and DBA (green). Bottom row shows statistically significant colocalization (white) of FN and ITGA8 in the nephrogenic zone of $FN^{+/+}$ kidneys by the use of ImageJ (V.1.45) and the colocalization finder algorithm by Christophe Laummonerie and Jerome Mutterer (Institut de Biologie Moleculaire des Plantes, Strasbourg, France).

Supplemental Figure 5. Colocalization of fibronectin and GDNF. Representative photos of metanephric mouse kidneys that were harvested at E13.5 and cultured *ex vivo* ± HT for five days. Thereafter, FN-competent kidneys ($FN^{+/+}$) and FN-deficient kidneys ($FN^{-/-}$) were stained for fibronectin (FN; magenta), GDNF (red), nuclei (DAPI; blue) and DBA (green). Bottom row shows statistically significant colocalization (white) of FN and GDNF in the nephrogenic zone of $FN^{+/+}$ kidneys by the use of ImageJ (V.1.45) and the colocalization finder algorithm by Christophe Laummonerie and Jerome Mutterer (Institut de Biologie Moleculaire des Plantes, Strasbourg, France).

Supplemental Figure 1

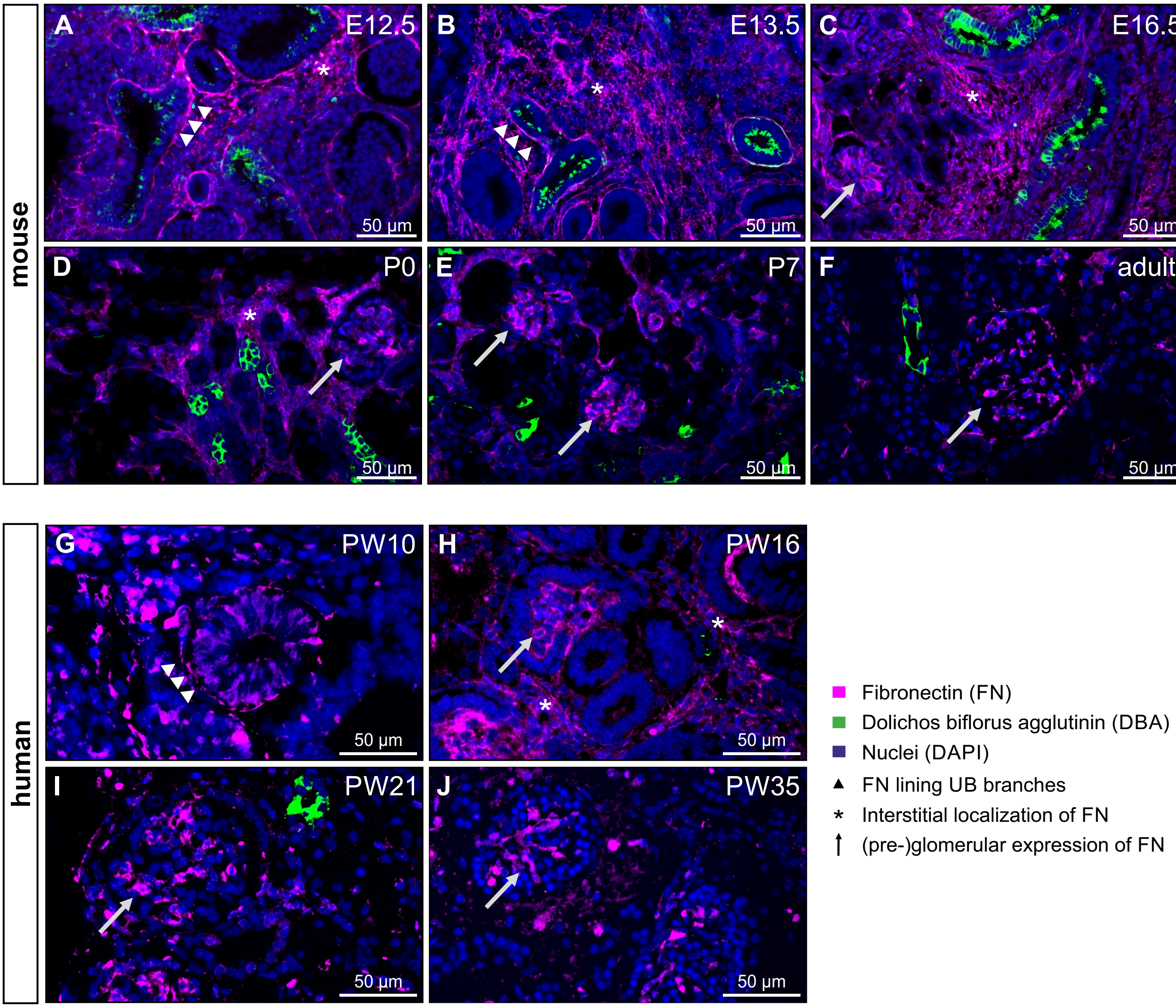

Supplemental Figure 2

Uncropped blots from figure 2C

MW (kDa)

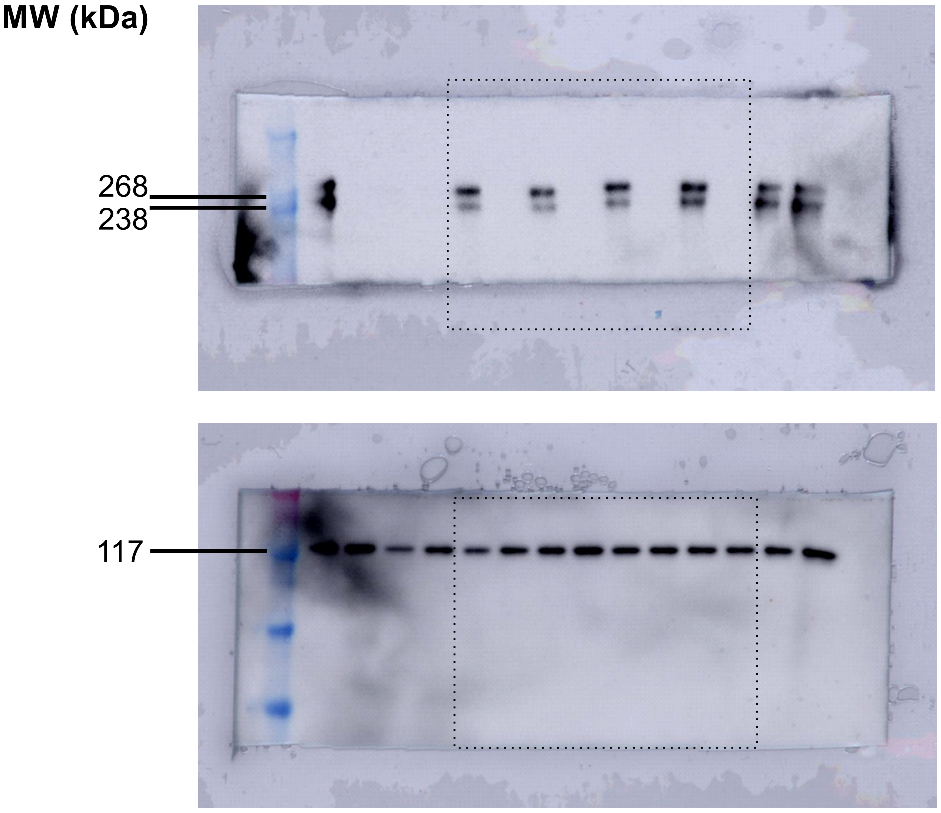

268
238
FN

117
Vinculin

Uncropped blots from figure 2D

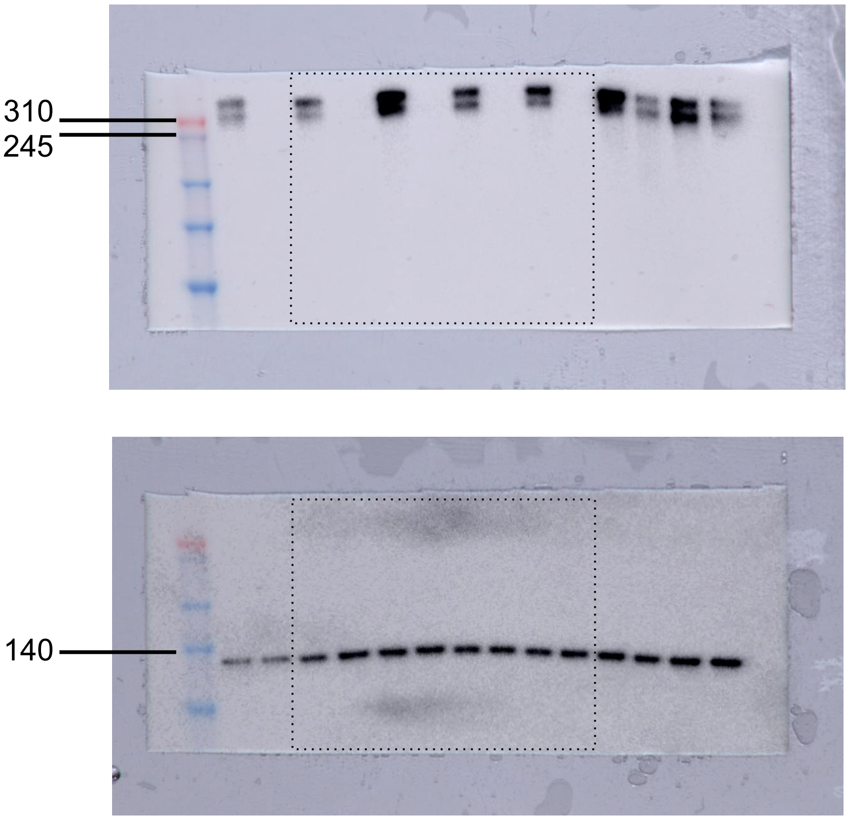

310
245
FN

140
Vinculin

Supplemental Figure 3

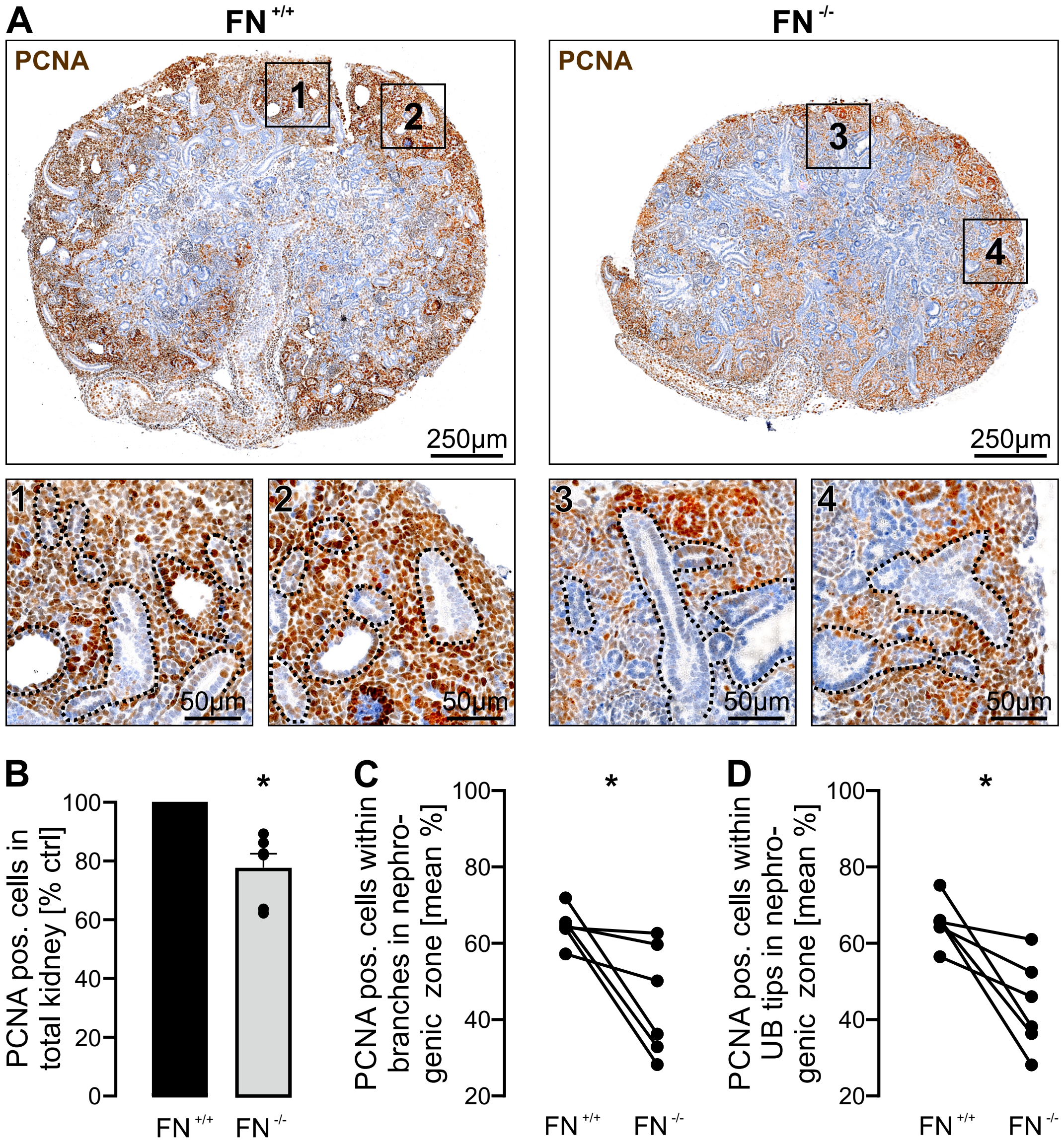

Supplemental Figure 4

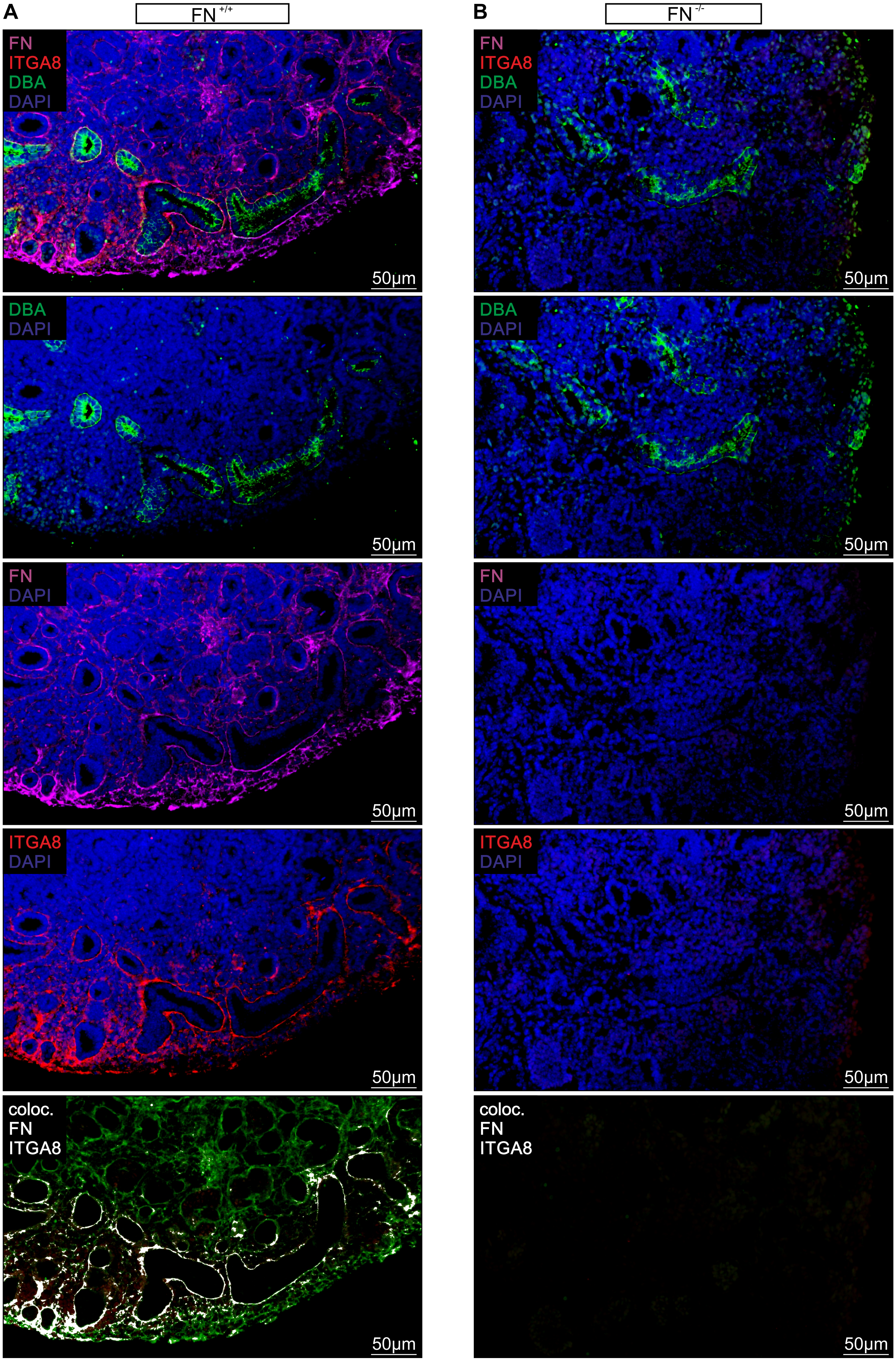

Supplemental Figure 5

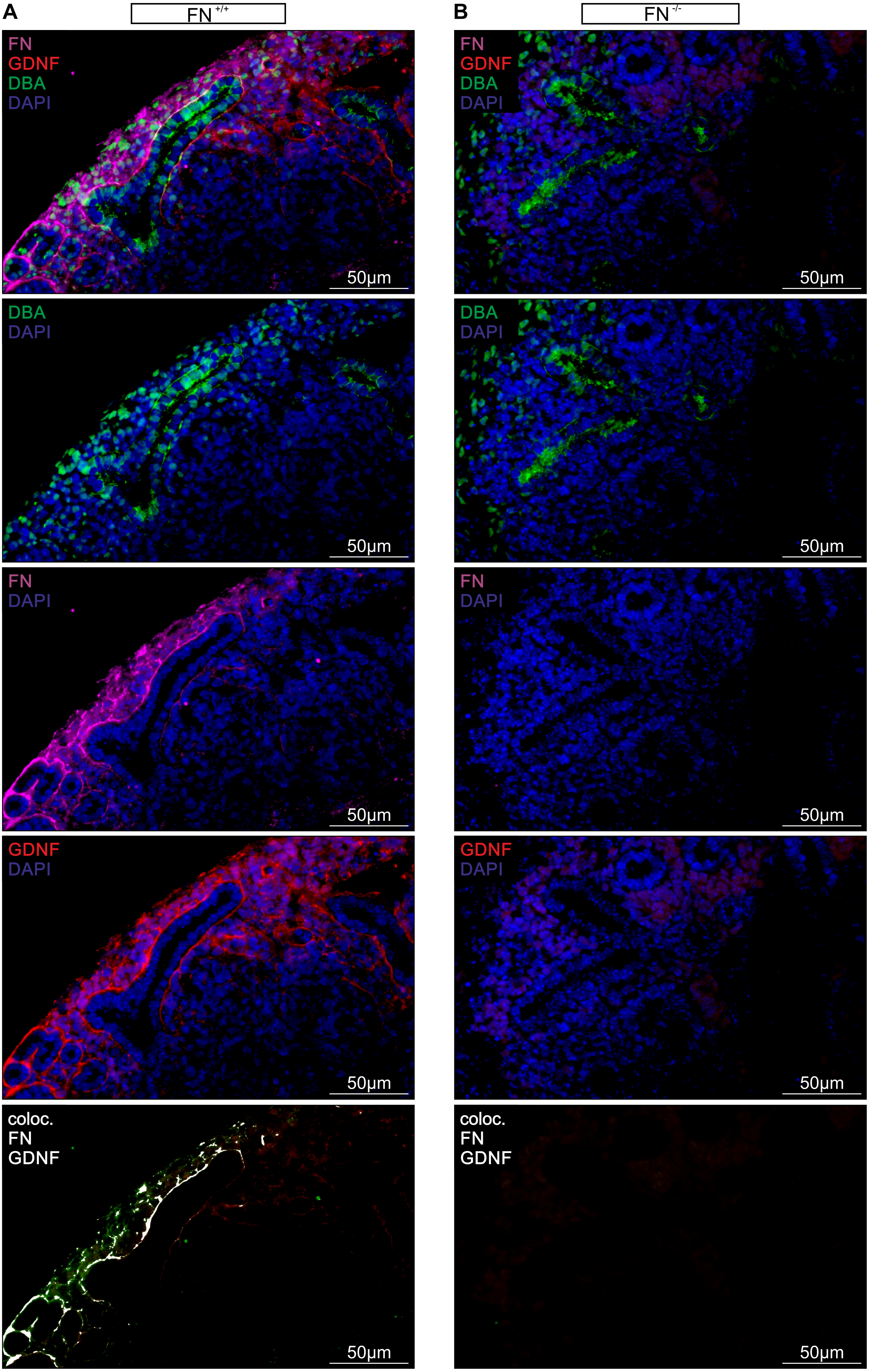

Supplement: Supplementary file 1 — Supplementary file1 (PDF 20465 KB) [file 424_2024_2954_MOESM1_ESM.pdf]
